# Vascular Consequences: A Case Report on Posterior Circulation Infarction as a Sequela of COVID-19

**DOI:** 10.7759/cureus.65837

**Published:** 2024-07-31

**Authors:** Anil M Philip, Lina J George, Shubhashis Saha, Sethu Sadanandan

**Affiliations:** 1 Internal Medicine, Kuriakose Chavara Memorial Hospital, Nooranad, IND; 2 Internal Medicine, John H. Stroger, Jr. Hospital of Cook County, Chicago, USA; 3 Radiodiagnosis, New Desinganad Scans, Kayamkulam, IND

**Keywords:** acute cerebrovascular accident, anticoagulation, posterior circulation stroke, sars-cov2, covid 19

## Abstract

This case report presents a posterior circulation infarction in a previously healthy 39-year-old male, three months post-severe COVID-19. He presented with right-sided homonymous hemianopia and elevated inflammatory markers and D-dimer levels. Imaging revealed an acute left occipital infarct. Such post-COVID-19 posterior circulation strokes are rare. This report discusses the pathophysiology, optimal anticoagulation therapy for COVID-19-related thrombotic complications, and early predictor models. This case underscores the need to recognize thromboembolic events as potential late sequelae in severe COVID-19 cases.

## Introduction

Severe acute respiratory syndrome-Coronavirus 2 (SARS-CoV2), first identified in China in late 2019, is associated with coronavirus disease 2019 (COVID-19), which commonly presents with fever, malaise, and respiratory symptoms [1]. The occurrence of vascular thrombotic complications after severe acute COVID-19 has been well-documented [2]. The mechanism of thrombus formation in COVID-19 has been best described as "immunothrombosis", secondary to the interaction between the viral pathogen and the host’s innate immune response [3,4]. We describe a young patient presenting with acute ischemic stroke to our healthcare facility three months after recovery from COVID-19 infection.

## Case presentation

A 39-year-old man, with no known comorbidity or relevant family history presented with a history of sudden-onset light-headedness, headache, and right-sided partial loss of vision. On examination, he was noted to have a hypertensive emergency, with a blood pressure of 210/150 mmHg, and central nervous system examination revealed a right-sided homonymous hemianopia with no other defects.

Three months prior, he had been admitted with severe SARS-CoV2 infection (COVID-19 reverse transcription polymerase chain reaction (RT-PCR) positive and WHO ordinal scale 6) requiring ICU admission and non-invasive mechanical ventilation. During the mentioned hospital stay, he was treated with remdesivir, prophylactic anticoagulation, and high-dose methylprednisolone, with which he had a full recovery.

**Figure 1:**
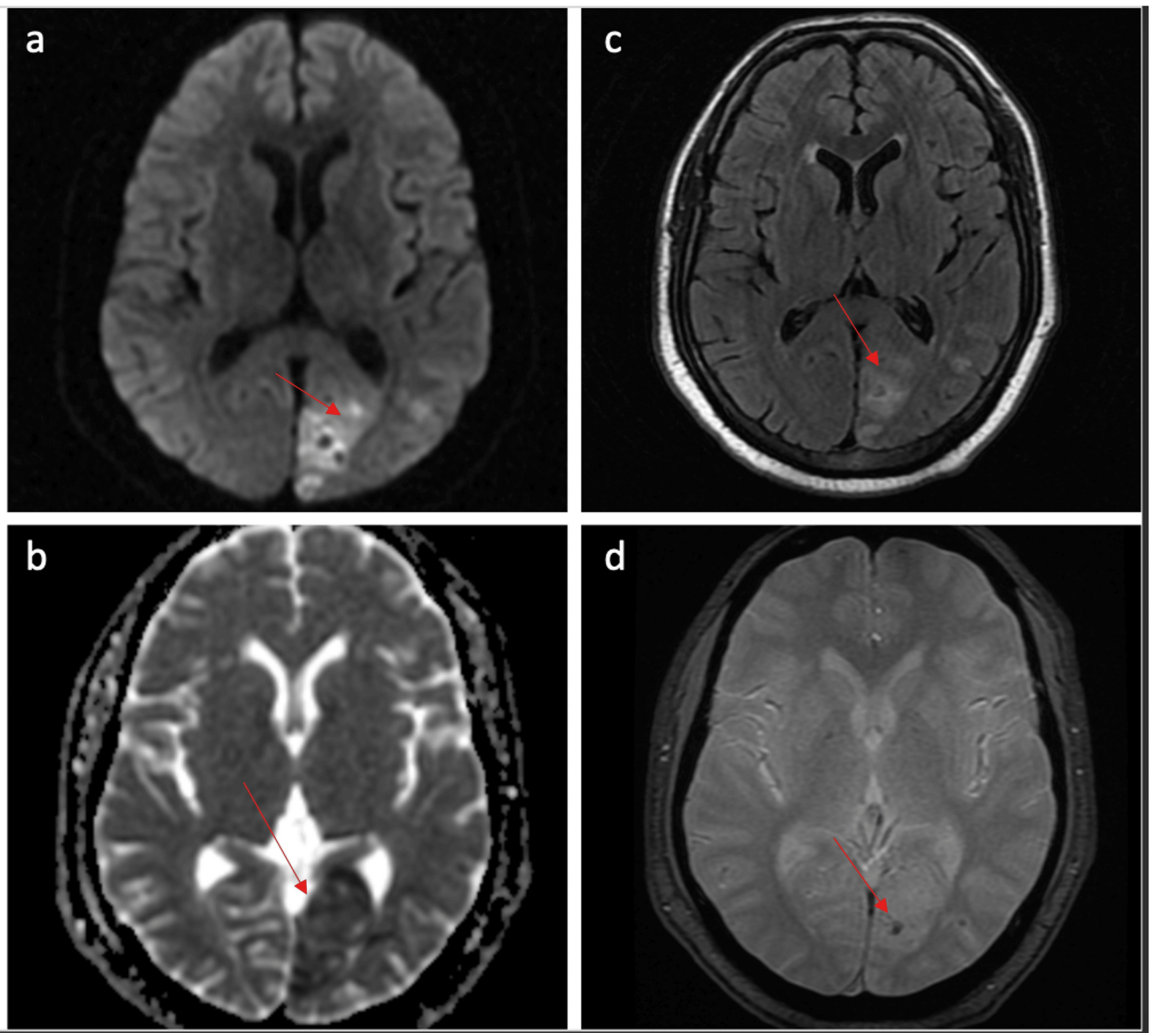
MRI brain a. Diffusion-weighted images(DWI) show an area of restricted diffusion in the left occipital lobe; b. Corresponding low value on apparent diffusion coefficient (ADC) mapping; c. The area is hyperintense on fluid-attenuated inverse recovery (FLAIR); d. Area of blooming on gradient echo (GRE) sequence consistent with microhemorrhage

During his current evaluation, a computed tomography (CT)was performed, and he was found to have an ill-defined hypodensity in the occipital region. Magnetic resonance imaging and angiography confirmed the presence of an acute infarct in the left occipital region with no large vessel occlusion (Figure 1). There was blooming on gradient echo sequences suggestive of microhemorrhage.

He was also noted to have elevated D-dimer and other inflammatory markers (Table 1).

**Table 1:** Laboratory investigations

	Current admission	Previous admission
Total white cell count (per mm^3^)	5,850	9,000
Differential count	N-64%, L-35%, E-1%	N-61%, L-38%, E-1%
D-dimer (ng/mL)	462.08	702.99
Ferritin (ng/mL)	3818.15	5988.2
LDH (IU/L)	996	1214
HbA1c	5.8%	-
Total cholesterol(mg/dl)	136	-
Triglyceride(mg/dl)	170	-
High-density lipoprotein(mg/dl)	26	-
Low-density lipoprotein(mg/dl)	76	-
Antinuclear antibody	Negative	-
Homocysteine (mmol/dl)	10.04 (5.46-16.2)	-
Phospholipid antibody IgM (U/ml)	3.9 (<15)	-
Phospholipid antibody IgG (U/ml)	3.2 (<15)	-

Cardiac evaluation was normal (Table 2).

**Table 2:** Imaging and other investigations MRA: magnetic resonance angiography; ACA: anterior cerebral artery; MCA: middle cerebral artery; PCA: posterior cerebral artery; RWMA: regional wall motion abnormalities; LVEF: left ventricular ejection fraction; HFA: Humphrey field analyzer

MRI Brain (Figure 1)	Acute infarct in the left occipital region with microhemorrhage (PCA territory)
MRA brain and neck	Bilateral normal ACA, MCA, and PCA, bilateral normal carotid and vertebral arteries, no anomalies of the posterior circulation
Echocardiogram	Normal heart chambers and valves, no RWMA, LVEF 70%, no clots or vegetation, no intracardiac shunts
24-hour Holter monitoring	Normal
Field analysis (HFA 30-2)	Right homonymous hemianopia

He was diagnosed to have post-COVID-19 sequela, hypertensive emergency with posterior circulation infarct (left occipital infarct) NIH Stroke Scale (NIHSS) -1. Therefore, he was initiated on dual antiplatelet therapy with aspirin and clopidogrel for 21 days, followed by single-agent aspirin.

## Discussion

Our case report describes a posterior circulation stroke due to a small vessel occlusion three months after a severe COVID-19 infection. This effect was not caused by cardioembolic or autoimmune causes. In patients with COVID-19, ischemic stroke appears to have an incidence of 1.8%, with a median time to development of stroke from the time of COVID-19 symptom onset reported as 16 days [5]. COVID-19-associated stroke patients tend to have higher NIHSS scores, greater incidence of cardioembolic etiology, and worse prognosis. Posterior circulation stroke is rare, with an incidence of 3.3% noted from a multicenter study in South India [6]. In a multinational study from 71 countries including 323 patients of ischemic stroke, patients presenting with bilateral visual field defects were seen in only two patients (0.006%) [7].

Cytokine storm-induced direct endothelial damage and hypercoagulability are attributed as the pathophysiology of stroke in COVID-19. Evidence suggests that the hypercoagulability of SARS-CoV-2 involves thrombo-inflammation triggered by viral infection originating in the pulmonary vasculature [8]. In patients with severe COVID-19 infection, the pulmonary thrombosis progresses to a systemic hypercoagulable state and widespread thrombosis.

The advantages and disadvantages of anticoagulation in preventing thromboembolic complications in COVID-19 are still debatable. A recent Italian multicenter study found increased anticoagulation failure in the setting of COVID-19 infection and stroke when compared to non-COVID-19-related ischemic strokes [9]. Though COVID-19, being a hyperinflammatory state with a higher incidence of disseminated intravascular coagulation and virus-induced vasculopathy, might pose a higher risk of hemorrhagic transformation, studies have shown that even recombinant plasminogen activators can safely be used in patients with ischemic strokes after COVID-19 infection [10]. Thus, anticoagulation decisions must consider hemorrhagic consequences and be patient-specific.

For hospitalized non-critically ill patients, therapeutic-dose heparin appears beneficial regardless of D-dimer results. However, in critically ill patients, it did not improve outcomes with a high probability of adverse events [11]. A systemic review and meta-analysis of studies on the thrombotic and bleeding risk associated with COVID-19 showed that thrombotic events occurred earlier during hospital admission than bleeding events (median 7·0 days (IQR 5.9-8.2) vs. 11.4 days (8.6-14.1) after admission) and the authors suggested avoiding extended duration, therapeutic-dose anticoagulation [12]. The MICHELLE trial concluded that patients who received standard heparin thromboprophylaxis in the hospital followed by 35 days of low-dose rivaroxaban post-discharge had a reduced risk of developing venous thrombo-embolic events as opposed to those who received heparin alone (3% as compared to 9%) [13]. However, there was no significant difference in the incidences of arterial thromboses, ischaemic stroke, or myocardial infarction. The International Medical Prevention Registry on Venous Thromboembolism and D‐Dimer score (IMPROVE‐DD) has been validated as a valuable tool to predict the occurrence of both in-hospital and post-discharge venous thrombosis. It can be used as a guide to prescribing extended prophylactic anticoagulation at discharge [14]. For patients experiencing acute post-COVID-19 thrombotic stroke of unknown etiology, guidelines recommend initiating dual antiplatelet therapy as in non-COVID-19 acute stroke cases [15].

## Conclusions

Thromboembolic events as a post-SARS-CoV2 infection sequela are well-established phenomena commonly seen as an early complication in patients. It is important to note that thromboembolic events may be seen as a late sequela in patients with a history of severe SARS-CoV2 infection, as seen in our patient. Prophylactic anticoagulation during hospital stay may prevent early thromboembolic events; later presentations should be treated on a need-to-treat basis. Current guidelines continue to recommend only dual antiplatelet therapy (DAPT) for the treatment of acute ischemic stroke of unknown cause post-COVID-19.
